# PIN1 in Cell Cycle Control and Cancer

**DOI:** 10.3389/fphar.2018.01367

**Published:** 2018-11-26

**Authors:** Chi-Wai Cheng, Eric Tse

**Affiliations:** Department of Medicine, The University of Hong Kong, Hong Kong, Hong Kong

**Keywords:** PIN1, phosphorylation, cell cycle, checkpoint, isomerization

## Abstract

Cell cycle progression is tightly controlled by many cell cycle-regulatory proteins that are in turn regulated by a family of cyclin-dependent kinases (CDKs) through protein phosphorylation. The peptidyl-prolyl *cis*/*trans* isomerase PIN1 provides a further post-phosphorylation modification and functional regulation of these CDK-phosphorylated proteins. PIN1 specifically binds the phosphorylated serine or threonine residue preceding a proline (pSer/Thr-Pro) motif of its target proteins and catalyzes the *cis*/*trans* isomerization on the pSer/Thr-Pro peptide bonds. Through this phosphorylation-dependent prolyl isomerization, PIN1 fine-tunes the functions of various cell cycle-regulatory proteins including retinoblastoma protein (Rb), cyclin D1, cyclin E, p27, Cdc25C, and Wee1. In this review, we discussed the essential roles of PIN1 in regulating cell cycle progression through modulating the functions of these cell cycle-regulatory proteins. Furthermore, the mechanisms underlying PIN1 overexpression in cancers were also explored. Finally, we examined and summarized the therapeutic potential of PIN1 inhibitors in cancer therapy.

## Introduction

Cell cycle involves a series of sequential events and is divided into several phases including protein synthesis in G1 phase, DNA synthesis in S phase, cell growth in G2 phase, chromosome segregation in mitosis and cell separation in cytokinesis. Regulation of these events depends on a series of phosphorylation and dephosphorylation of various cell cycle-regulatory proteins. A family of cyclin-dependent kinases (CDKs) has been found to play an important role in regulating cell cycle events through phosphorylation of their substrates in serine or threonine residues preceding proline (pSer/Thr-Pro) motif. In addition, the peptidyl-prolyl *cis*/*trans* isomerase PIN1 has been identified to provide a further post-phosphorylation modification and functional regulation of these CDK-phosphorylated proteins during cell cycle progression (Lu, 2000). PIN1 specifically binds the pSer/Thr-Pro motif of its target proteins through its N-terminal WW binding domain and catalyzes *cis*/*trans* isomerization of the pSer/Thr-Pro peptide bonds with its C-terminal prolyl isomerase (PPIase) domain (Lu et al., 1999; Zhou et al., 2000; Behrsin et al., 2007). Through isomerization, PIN1 induces conformational changes of its bound proteins, thereby regulating their cellular functions, protein stability, subcellular localization, potential interactions with other proteins, and further phosphorylation/dephosphorylation status.

The first evidence showing the role of PIN1 in regulating cell cycle progression is the discovery of its interaction with NIMA (Never In Mitosis), a fungal mitotic-regulatory protein kinase (Lu et al., 1996). PIN1 over-expression has been found to arrest cells in G2 phase and delay cell entry into mitosis, suggesting that PIN1 negatively regulates cell cycle progression (Lu et al., 1996; Shen et al., 1998). PIN1 also binds to many cell cycle-regulatory proteins including Cdc25C, cyclin D1, cyclin E, Myt1, p27, and Wee1 (Shen et al., 1998; Wells et al., 1999; Stukenberg and Kirschner, 2001; Liou et al., 2002; Yeh et al., 2006; Okamoto and Sagata, 2007; Zhou et al., 2009). Through PIN1-catalyzed isomerization, PIN1 fine-tunes the cellular functions of these cell cycle-regulatory proteins. Given the importance of PIN1 in cell cycle progression, it is expected that PIN1 may be implicated in cancer development. Indeed, PIN1 over-expression has been found in many cancers and it promotes uncontrolled cell proliferation and malignant cell transformation in different cancer models. In addition to cancer, PIN1 also plays a critical role in other human diseases. PIN1 is essential for stabilization of cytokine GM-CSF and TGF-β1 mRNAs and eosinophil survival (Shen et al., 2005, 2008). As activation of eosinophils causes inflammation, it has been proposed that PIN1 regulates inflammatory responses in diseases such as asthma. Furthermore, increased PIN1 expression and activity have been found in aortas of diabetic mice and peripheral blood monocytes of type 2 diabetes mellitus patients (Paneni et al., 2015). In response to high glucose levels, PIN1 expression is increased in human endothelial cells. PIN1 enhances pancreatic β-cell proliferation and insulin secretion. On the other hand, PIN1 also increases reactive oxygen species (ROS) generation in vascular endothelial cells through its interaction with pro-oxidant adaptor p66^Shc^. Increased ROS production in turn leads to oxidative damage to vascular endothelium. Therefore, it is speculated that PIN1 may be involved in the development of diabetes and diabetic vascular complications.

In this article, the essential role of PIN1 in cell cycle regulation, the mechanisms underlying deregulated PIN1 expression in cancer, and the therapeutic potential of PIN1 inhibitors in cancer therapy were discussed.

## PIN1 is Essential for Cell Cycle Progression

**FIGURE 1:**
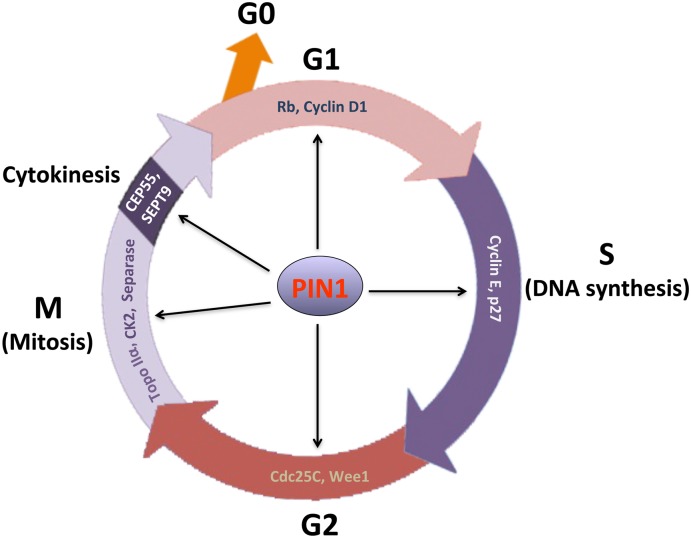
Essential role of PIN1 in regulation of cell cycle progression. Through phosphorylation-dependent prolyl isomerization, PIN1 regulates the functions of various cell cycle-regulatory proteins for cell cycle progression. To mediate the progression of G1 checkpoint, PIN1 activates cyclin D1-CDK4/6 through inactivation of retinoblastoma protein (Rb) and accumulation of cyclin D1 protein. PIN1 is able to promote G1-S phase transition by increasing CDK2 activity in spite of its paradoxical regulation of cyclin E and p27 levels. In G2 phase, PIN1 functions to regulate cyclin B-CDK1 activity through its modulation of the activity of Cdc25C protein phosphatase and Wee1 kinase. In addition, PIN1 regulates chromosome condensation and segregation through its interaction with topoisomerase (Topo) IIα and casein kinase (CK) 2, and separase, respectively. During cytokinesis, PIN1 alters the activities of centrosomal protein (CEP55) and septin 9 (SEPT9) that function to mediate midbody abscission. Consequently, PIN1 is required for cell cycle progression.

Although PIN1 was first identified as a mitotic regulator, it has also been shown subsequently to regulate the functions of various phosphorylated proteins in other phases of the cell cycle (Figure 1). Among the four distinct phases in the cell cycle, the progression to G1 phase is the most critical step for cell proliferation. In a resting cell at G0 phase, retinoblastoma protein (Rb) is hypophosphorylated and binds to the E2F family of transcription factors. E2F family proteins are transcriptional activators of the genes encoding cell cycle-regulatory proteins, including cyclin A, cyclin E, cyclin-dependent kinase (CDK) 2 and Cdc25. The binding of Rb to E2F proteins prevents the release of E2F proteins into the nucleus, thereby inhibiting the entry of cell cycle. As a result, the cell will remain in the resting quiescent state. On the other hand, in order to start cell proliferation, the dissociation of E2F family is required for the cell to pass through the G1 checkpoint (restriction point) and to proceed with DNA synthesis in the S phase. In the early G1 phase, activation of the CDK by cyclin D1 is the major regulatory event for the cell to proceed in the G1 checkpoint. Upon binding to cyclin D1, CDK4/6 kinase is activated to induce phosphorylation of Rb (Nevins, 2001; Giacinti and Giordano, 2006). The CDK-mediated Rb phosphorylation leads to the dissociation of E2F from Rb, resulting in increased E2F transcriptional activity. As a result, transcriptional activation of E2F triggers the expression of cell cycle regulatory proteins and promotes cell cycle progression through the G1 checkpoint.

### PIN1 Promotes G1 Checkpoint Progression

Because cyclin D1 is a major regulator of the G1 checkpoint progression, deregulation of cyclin D1 would lead to uncontrolled cell proliferation and tumor formation. Over-expression of cyclin D1 is commonly found in various types of cancers including breast, colon, liver and lung cancers (Gillett et al., 1994; Nishida et al., 1994; Keum et al., 1999; Mermelshtein et al., 2005). Interestingly, PIN1 expression is positively correlated with cyclin D1 expression in these cancers (Wulf et al., 2001; Nakashima et al., 2004; Pang et al., 2004). Cyclin D1 expression has been shown to be regulated by PIN1 at the transcriptional and translational levels. At the transcriptional level, PIN1 binds the phosphorylated Thr246-Pro motif of β-catenin to prevent cytoplasmic translocation and protein degradation of β-catenin (Ryo et al., 2001). This results in the stabilization and transcriptional activation of β-catenin, which in turn enhances cyclin D1 expression. Through its interaction with the phosphorylated Ser63/73-Pro motif of c-Jun and the phosphorylated Thr254-Pro motif in the p65/RelA subunit of NF-κB, PIN1 has also been shown to increase the c-Jun and NF-κB transactivation activity toward *cyclin D1* gene (Wulf et al., 2001; Ryo et al., 2003). At the translational level, PIN1 directly binds to the phosphorylated Thr286-Pro motif of cyclin D1 to increase cyclin D1 stability and nuclear accumulation. These findings suggest that PIN1 over-expression increases cyclin D1 expression to promote cell cycle progression through the release of E2F transcription factors from phosphorylated Rb. Recently, we have also reported cell cycle arrest at G0/G1 phase in PIN1-depleted cells (Cheng et al., 2017), further confirming the importance of PIN1 expression in promoting the exit of cells from G1 checkpoint.

**FIGURE 2:**
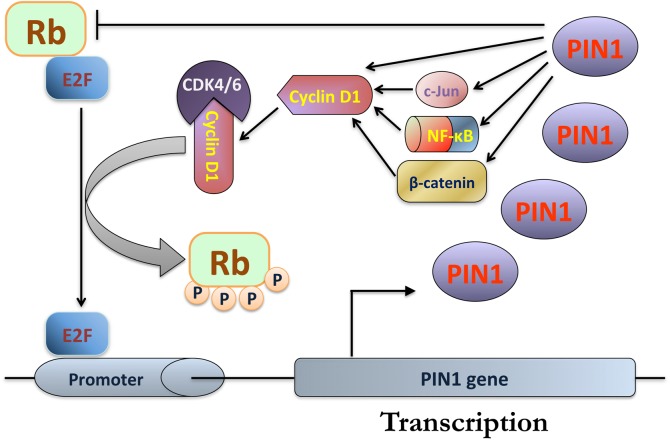
PIN1-mediated positive feedback loop for progression of G1 checkpoint. During G1 phase, PIN1 increases the binding of retinoblastoma protein (Rb) with cyclin D1-CDK4/6, resulting in inactivation of Rb and the release of E2F for the activation of PIN1 expression. Through PIN1-mediated isomerization, PIN1 enhances cyclin D1 expression by increasing the transcriptional activities of β-catenin, nuclear factor kappa B (NF-κB) and c-Jun. In addition, PIN1 also binds and increases the protein stability of cyclin D1, which in turn enhances its protein level. As a result, cyclin D1 binds and activates CDK4/6, leading to further inactivation of Rb for E2F dissociation.

In addition to regulating cyclin D1 expression, PIN1 has been found to interact directly with phosphorylated Rb and to increase its binding with cyclin D1-CDK4/6, promoting the dissociation of E2F transcription factors (Rizzolio et al., 2012; Tong et al., 2015). Interestingly, PIN1 is also one of the transcriptional targets of E2F family. The dissociated E2F binds to the E2F-binding sites of the *PIN1* gene promoter to activate PIN1 expression (Ryo et al., 2002). Therefore, it is speculated that there is a PIN1-mediated positive feedback loop for G1 checkpoint progression through the regulation of Rb phosphorylation and E2F dissociation (Figure 2). Several studies have provided direct and indirect evidence to support the existence of this positive feedback loop. Firstly, *Pin1*-null (*Pin1^-/-^*) mouse embryonic fibroblasts (MEFs) exhibit reduced cyclin D1 expression and Rb phosphorylation while reconstitution of PIN1 expression in *Pin1^-/-^* MEFs restores cyclin D1 expression and Rb phosphorylation (You et al., 2002). In addition, a positive correlation between PIN1 and Rb phosphorylation has been shown in human glioma and breast tumor tissues (Rizzolio et al., 2013; Tong et al., 2015). Furthermore, Tong et al. (2015) has demonstrated that down-regulation of PIN1 increases the binding of Rb with E2F. Through this positive feedback loop, PIN1 may contribute to the G1 checkpoint progression through phosphorylation of Rb and over-expression of PIN1 and cyclin D1 in cancers.

### PIN1 Regulates G1-S Phase Transition

After initial Rb phosphorylation by cyclin D1-CDK4/6 complex, a second wave of Rb phosphorylation is mediated by cyclin E-CDK2 complex. Cyclin E is one of the E2F transcriptional targets and its expression occurs after the release of E2F from the first wave of cyclin D1-CDK4/6-mediated Rb phosphorylation. As a result, enhanced cyclin E expression increases cyclin E-CDK2 activity, promoting cell entry into the S phase (Resnitzky et al., 1994). In addition to cyclin E binding, CDK2 activity is also regulated by its inhibitor p27. p27 inhibits CDK2 at G1 phase and prevents premature entry into S phase. On the other hand, cyclin E-CDK2 also phosphorylates p27 on Thr187, targeting the phosphorylated p27 to SCFskp2 ubiquitin ligase for protein degradation (Carrano et al., 1999; Ganoth et al., 2001). Therefore, the increase in cyclin E expression by Rb phosphorylation results in CDK2 activation and subsequent p27 protein degradation. Finally, the decrease in p27 level further enhances CDK2 activity to promote the G1-S phase progression by reducing its inhibitory effect on CDK2.

PIN1 interacts with the CDK2 regulators, cyclin E and p27, and their interactions with PIN1 are dependent on the CDK2-mediated phosphorylation of their PIN1-binding motifs. PIN1 binds the phosphorylated Ser384-Pro motif of cyclin E and enhances its protein degradation. In addition, PIN1 also binds the phosphorylated Thr187-Pro motif on p27 and inhibits its protein degradation (Yeh et al., 2006; Zhou et al., 2009). As a result, PIN1 expression results in decreased CDK2 positive regulator cyclin E but increased CDK2 inhibitor p27. Despite its effect on decreasing cyclin E and increasing p27 levels, over-expression of PIN1 has been shown to promote cell proliferation in different cell types. To explain this apparent paradoxical phenomenon, a recent study by our group has demonstrated that PIN1 relieves CDK2 inhibition by regulating the CDK2-inhibitory activity of p27. We showed that PIN1 expression not only stabilizes p27, but also reduces its binding affinity with cyclin E-CDK2, thereby impairing its CDK2-inhibitory activity. Therefore, PIN1 expression results in increased total CDK2 (cyclin E- and cyclin A-CDK2) and cyclin E-CDK2 activities in spite of its associated decrease in cyclin E and increase in p27 levels. As a result, PIN1-mediated increase in CDK2 activities enhances the proportion of S phase proliferating cells and promotes cell proliferation. Although the mechanism underlying the catalytic role of PIN1 in altering the p27 binding affinity with cyclin E-CDK2 is unclear, these findings clearly reveal the important role of PIN1 in maintaining CDK2 activities and thereby promoting G1-S phase transition.

### PIN1 is Essential for a Normal G2-M Phase Transition

PIN1 functions as a mitotic regulator through its interaction with many mitotic phosphorylated proteins including Cdc25C, Wee1, Polo-like kinase 1 (PLK1), and Myt1 at G2-M phase transition (Shen et al., 1998). To mediate the progression of cells at the G2-M phase transition, a progressive activation of CDK1 is required through binding with cyclin B and subsequent dephosphorylation of CDK1 subunit at Thr14 and Tyr15. These events result in complete activation of the cyclin B-CDK1 complex which in turn initiates mitosis. The protein phosphatase Cdc25C is responsible for the dephosphorylation of CDK1 subunit at Thr14 and Tyr15, resulting in activation of cyclin B-CDK1. Cdc25C phosphatase activity is regulated by PLK1. PLK1 phosphorylates Cdc25C to promote its nuclear translocation, leading to increased Cdc25C phosphatase activity toward Thr14 and Tyr15 sites of the CDK1 subunit (Toyoshima-Morimoto et al., 2002). In addition to PLK1, PIN1 has also been found to play an important role in regulating Cdc25C phosphatase activity although the exact mechanism remains to be elucidated. PIN1 is originally found to induce Cdc25C dephosphorylation and inhibits its phosphatase activity, resulting in inhibition of cyclin B-CDK1 activation (Crenshaw et al., 1998; Shen et al., 1998; Zhou et al., 2000). However, another study by Stukenberg and Kirschner has demonstrated an opposing effect of PIN1 in regulating Cdc25C phosphatase activity (Stukenberg and Kirschner, 2001). PIN1 can either decrease or increase the Cdc25C activity when incubates with different proteins in *Xenopus* extract cell-free system. Similar to the previous findings, PIN1 can inhibit Cdc25C phosphatase activity in the presence of active CDK1 kinase. Moreover, PIN1 has no effect on Cdc25C phosphatase activity in the presence of Plx1 (*Xenopus* PLK1 ortholog). Interestingly, PIN1 increases Cdc25C phosphatase activity in the presence of both CDK1 and Plx1 kinases. Therefore, it is speculated that the differential phosphorylation of Cdc25C by CDK1 and Plx1 kinases may affect its phosphatase activity through PIN1 binding and isomerization.

On the other hand, two mitotic protein kinases, Wee1 and Myt1, have been found to induce the phosphorylation of CDK1 subunit at Thr14 and Tyr15, resulting in inhibition of cyclin B-CDK1 activity and preventing cell mitosis prematurely. In fact, the cyclin B-CDK1 complex also phosphorylates Wee1 at Thr186-Pro motif, facilitating the binding between PIN1 and Wee1 (Okamoto and Sagata, 2007). As a result, PIN1 binds Wee1 and impairs its kinase activity toward Thr14 and Tyr15 sites in the CDK1 subunit. Thus, impairing Wee1 activity by PIN1 enhances cyclin B-CDK1 activity, allowing cell entry into mitosis. In addition to Cdc25C and Wee1, PIN1 also binds Myt1 and PLK1. However, the binding does not exert any regulatory effect on the activation of CDK1 kinase (Wells et al., 1999; Eckerdt et al., 2005). Through its interaction with Cdc25C and Wee1, PIN1 therefore modulates the cyclin B-CDK1 activity for the initiation of mitosis.

In mitosis, chromosome condensation is the essential step for the proper segregation of sister chromatids. PIN1 has been found to mediate chromosome condensation through its interaction with mitotic topoisomerase (Topo) IIα (Xu and Manley, 2007). Both PIN1 and phosphorylated Topo IIα are colocalized on mitotic chromosomes and over-expression of PIN1 increases Topo IIα phosphorylation. Topo IIα phosphorylation regulates its activity for chromosome condensation. However, PIN1 only promotes chromosome condensation with the presence of both Topo IIα and cyclin B-CDK1 while PIN1 or cyclin B-CDK1 alone has no effect on chromosome condensation by incubating with Topo IIα. Therefore, it is suggested that overexpressing PIN1 together with cyclin B-CDK1 regulates chromosome condensation through its interaction with Topo IIα. Casein kinase (CK) 2 is another kinase that regulates the phosphorylation and activity of Topo IIα to mediate chromosome condensation. In fact, PIN1 has been shown to regulate CK2 localization in the mitotic spindle and thus inhibit its kinase activity toward Topo IIα (Messenger et al., 2002; St-Denis et al., 2011). Taken together, these findings suggest that PIN1 is essential in coordinating the chromosome condensation during mitosis.

During chromosome condensation, the paired sister chromatids are held together by cohesin protein. To cleave the cohesin protein, the protease activity of separase is required for the segregation of the paired sister chromatids. The separase activity is tightly regulated to control a proper segregation of sister chromatids. Separase is inactivated when binding with cyclin B-CDK1 or securin. Before chromatids segregation, PIN1 has been found to bind the phosphorylated Ser1153-Pro motif of separase and enhance the binding affinity of separase with cyclin B-CDK1 (Hellmuth et al., 2015). Therefore, PIN1 inhibits the premature chromatids segregation by enhancing the inhibitory binding of cyclin B-CDK1 to separase. Securin is another binding partner of separase and their binding also inhibits separase activity. To initiate the chromatids segregation, anaphase promoting complex/cyclosome (APC/C) ubiquitin ligase triggers the proteasome degradation of securin and release of separase. Through PIN1-catalyzed isomerization, PIN1 renders previously securin-associated separase to be resistant against the inhibitory effect of free securin. However, PIN1 still enhances the binding of those PIN1-isomerized securin-resistant separase with cyclin B-CDK1. Consequently, most separase is inactivated by cyclin B-CDK1 binding. In parallel, phosphorylation of cyclin B-CDK1 reduces its binding with separase, resulting in the release of active separase. Therefore, the released separase cleaves cohesin to promote chromatids segregation. In short, the interaction between PIN1 and separase allows a rapid release of free separase for chromatids segregation and this regulatory mechanism occurs much faster than the regulation of securin by proteasome degradation. A rapid activation of separase is required to prevent delay or early chromatids segregation that results in chromosome instability. Therefore, the PIN1-mediated post-translational regulation is essential for the regulation of separase activity and chromatids segregation.

### Functional Role of PIN1 in Regulation of Cytokinesis

After mitosis, cytokinesis takes place to completely separate the parental cell into two daughter cells. During cytokinesis, PIN1 has been found to play an important role in mediating the midbody abscission process. Upon PIN1 depletion, cells exhibits defects in midbody abscission and a delay in cytokinesis (van der Horst and Khanna, 2009; Estey et al., 2013). The regulation of cytokinesis by PIN1 depends on its interaction with two cytokinesis-regulatory proteins, centrosomal protein (CEP55) and septin 9 (SEPT9). CEP55 functions as an abscission factor and its activity is inhibited by PLK1 phosphorylation (Bastos and Barr, 2010). PLK1-mediated phosphorylation of CEP55 prevents its localization to the midbody ring and thereby inhibits abscission. During cytokinesis, PIN1 interacts with CEP55 via its phosphorylated Ser425-Pro and Ser428-Pro motifs. Through this interaction, PIN1 maintains the PLK1-mediated phosphorylation state of CEP55 to inhibit its translocation to midbody ring and prevent premature abscission. In addition, SEPT9 is also crucial for proceeding midbody abscission as SEPT9-depleted cells exhibit abscission defects (Estey et al., 2010). Upon mitotic entry, PIN1 not only binds SEPT9 via its phosphorylated Thr24-Pro motif, but also regulates the SEPT9 function to mediate midbody abscission. Over-expression of PIN1-binding defective SEPT9 mutant results in abscission defects, suggesting that the PIN1 and SEPT9 interaction is indispensible for the completion of abscission and cytokinesis. Therefore, it is speculated that PIN1 deregulation may result in cytokinesis defects that lead to an increased genomic instability and malignant cell transformation through modulating the activities of CEP55 and SEPT9.

## PIN1 Regulates DNA Damage-Induced Cell Cycle Checkpoint

DNA damage occurs in cells as they progress through the cell cycle. In response to DNA damage, p53 plays a critical role in mediating cell cycle arrest or apoptosis to prevent the replication of the damaged DNA during G1-S phase transition or to halt progression into mitosis. DNA damage activates Ataxia Telangiectasis mutated (ATM) kinase, which in turn, phosphorylates checkpoint kinase (Chk)2. Subsequently, activated Chk2 phosphorylates p53 on Ser20 to reduce the interaction of p53 with MDM2 (Hirao et al., 2000). MDM2 functions as the E3 ubiquitin ligase to degrade p53 and the transcriptional repressor to inhibit p53 transactivation activity. As a result, the ATM/Chk2-dependent phosphorylation of p53 increases p53 protein stability and transactivation activity toward CDK inhibitor *p21* gene and several proapoptotic BCL2 family genes. Inhibition of CDK activity by p21 induces cell cycle arrest for DNA damage repair while activation of caspases by proapoptotic BCL2 family proteins, e.g., BAX and PUMA induces apoptosis for the elimination of damaged cell (Fischer, 2017). Therefore, p53 is the key regulator of cell fate decision in response to DNA damage.

PIN1 has been found to fine-tune the p53 functions in response to DNA damage during cell cycle progression. DNA damage increases the interaction between PIN1 and phosphorylated p53 at the Ser33-Pro, Ser46-Pro, Thr81-Pro, and Ser315-Pro motifs of p53 (Wulf et al., 2002; Zacchi et al., 2002; Berger et al., 2005). Through PIN1-catalyzed isomerization, PIN1 facilitates the interaction between p53 and Chk2 and enhances the phosphorylation of p53 at Ser20 by Chk2 in response to DNA damage. Phosphorylation of p53 at Ser20 reduces its interaction with its negative regulator MDM2, leading to an increase in p53 protein stability and transactivation activity (Wulf et al., 2002; Zheng et al., 2002; Berger et al., 2005). Therefore, in response to DNA damage, PIN1-expressing cells undergo cell cycle arrest by increasing p53 transactivation activity and CDK inhibitor p21 protein level while *Pin1*-null cells show defects in cell cycle arrest. All these findings suggest that PIN1 modulates p53 functions to induce cell cycle checkpoints in response to DNA damage.

## PIN1 Over-Expression in Cancer

During cell cycle progression, PIN1 is involved in the modulation of different functions of its interacting proteins through PIN1-mediated isomerization. Therefore, it is expected that PIN1 expression level is crucial for the regulation of cell cycle progression. Indeed, PIN1 over-expression has been found to promote cell proliferation and malignant cell transformation. Several studies including those from our group have demonstrated that PIN1 over-expression results in increased cell proliferation in hepatocellular carcinoma (HCC) cells (Pang et al., 2006, 2007; Cheng et al., 2013). Suizu et al. (2006) has also showed that PIN1 over-expression enhances the colony formation ability in soft agar and thereby contributes to cell transformation. In addition, a positive association between PIN1 expression and tumor progression is further validated in various types of human cancers including brain, breast, cervical, colon, liver and prostate (Wulf et al., 2001; Ayala et al., 2003; Bao et al., 2004; Pang et al., 2004). As a result, deregulation of PIN1 expression through different mechanisms has been studied in cancer. As discussed, PIN1 is one of the transcriptional targets of the E2F family and its expression is regulated by the Rb-E2F pathway. Because the Rb-E2F pathway is commonly deregulated in many cancers, it appears that abnormalities of this pathway may result in PIN1 overexpression and malignant transformation in cancer cells. Furthermore, PIN1 is also a direct transcriptional target of NOTCH1 and NOTCH4 Intracellular Domains (ICDs) (Rustighi et al., 2009, 2014). PIN1 has been found to interact with NOTCH1 and NOTCH4 ICDs and inhibits their proteasome-dependent degradation through reducing their interaction with the E3 ubiquitin-ligase Fbxw7α (Rustighi et al., 2014). Therefore, PIN1 expression stabilizes and increases NOTCH1 and NOTCH4 ICDs, which in turn, increases the transcription of *PIN1*, resulting in a positive feedback loop for deregulated PIN1 expression in cancers.

Small non-coding RNAs, microRNAs (miRNAs), provide another post-transcriptional mechanism in regulating PIN1 expression. In general, miRNA negatively regulates the gene expression by binding to the 3’UTR of its target mRNA (Zhang et al., 2006). As a result, suppression of PIN1-targeting miRNAs contributes to PIN1 over-expression in various cancers. The relationship between miRNAs and PIN1 expression was first demonstrated in breast cancer with miR-200b shown to be down-regulated and inversely correlated with PIN1 expression in breast tumor tissues (Zhang et al., 2013). In addition, several PIN1-targeting miRNAs that show a negative correlation with PIN1 expression have also been identified in other cancers. These cancer-associated miRNAs include miR-296-5p in prostate cancer (Lee et al., 2014), miR-874-3p and miR-140-5p in HCC (Leong et al., 2017; Yan et al., 2017), and miR-370 in esophageal squamous-cell carcinoma (Chen et al., 2018). Taken together, these PIN1-targeting miRNAs are down-regulated in many cancers, resulting in PIN1 over-expression.

## Development of PIN1 Inhibitors for Cancer Treatment

Numerous studies have demonstrated that depletion of PIN1 not only reduced cell proliferation, but also enhanced cellular apoptosis *in vitro* and induced tumor shrinkage in various types of cancer cells *in vivo*. Ryo et al. have shown that inhibition of PIN1 by over-expression of dominant-negative PIN1 mutant reduced cell proliferation and reversed transformed phenotypes on human breast epithelial cells while suppression of PIN1 expression by RNA interference reduced tumourigenicity and angiogenesis in xenograft mouse model of prostate cancer (Ryo et al., 2002, 2005). Our previous studies have also shown that PIN1 depletion in HCC cells inhibited tumor growth and enhanced tumor apoptosis *in vivo* (Pang et al., 2006; Cheng et al., 2013). Therefore, PIN1 inhibitors are attractive therapeutic agents for the treatment of cancers (Table 1).

**Table 1:** Potential PIN1 inhibitors for cancer treatment.

Drug	Details	Mechanisms of action	Tested models	Limitations
Juglone	First PIN1 inhibitor; Induces PIN1 protein degradation but a high juglone concentration is required; Inhibits tumor growth of prostate cancer	Irreversible inhibition of PIN1 PPIase catalytic domain	Cell line; Mouse	Non-specific inhibition of RNA polymerase II and Rab4
PiB	Inhibits colon cancer cell proliferation	Competitive inhibition of PIN1 PPIase catalytic domain	Cell line	Poor solubility in DMSO
Dipentamethylene thiuram monosulfide (DTM)	Inhibits colon cancer cell proliferation	Competitive inhibition of PIN1 PPIase catalytic domain	Cell line	No testing in animal model
TME-001	Inhibits cervical cancer cell proliferation	Competitive inhibition of PIN1 PPIase catalytic domain	Cell line	No testing in animal model
5′-nitro-indirubinoxime (5′-NIO)	Inhibits lung cancer cell proliferation; Causes G1 phase arrest	Inhibition of PIN1 activity in a concentration-dependent manner	Cell line	Non-specific inhibition of PLK1
Selenium derivative	Inhibits breast cancer stem cell proliferation	Specific inhibition of PIN1 PPIase catalytic domain	Cell line	No testing in animal model
All-*trans* retinoic acid (ATRA)	FDA^1^ approved for treatment of APL; Inhibits breast cancer and APL cell proliferation; Enhances cytotoxic effect of sorafenib in HCC^2^ model	Specific inhibition of PIN1 PPIase catalytic domain Degradation of PIN1 protein	Cell line; Mouse; Human (APL^3^ patient)	Short half-life (45 min)
Arsenic trioxide (ATO)	FDA^1^ approved for treatment of APL; Inhibits breast cancer cell proliferation and xenograft tumor growth	Specific inhibition of PIN1 PPIase catalytic domain by a non-covalent mechanism Degradation of PIN1 protein	Cell line; Mouse	Lack of PIN1 specificity Inducing protein degradation of cyclin D1 and NPM-ALK
KPT-6566	Exerts both PIN1-inhibitory and cytotoxic effects; More specific PIN1-inhibitory effect than PiB; Reduces lung metastasis with breast cancer cells *in vivo*	Specific inhibition of PIN1 PPIase catalytic domain Degradation of PIN1 protein	Cell line; Mouse	No testing in clinical trial
liposomal/cyclodextrin complex	Encapsulates potent PIN1 inhibitor; Increases water solubility of PIN1 inhibitor; Inhibits ovarian cancer cell proliferation and xenograft tumor growth	Liposomal delivery of PIN1 inhibitor to tumor tissue	Cell line; Mouse	No testing in clinical trial


*^1^FDA, the Food and Drug Administration agency from the United States; ^2^HCC, hepatocellular carcinoma; ^3^APL, acute promyelocytic leukemia.*

The first identified compound that inhibits PIN1 activity is juglone. Juglone has been demonstrated to inhibit PIN1 activity and suppress cell proliferation in various types of cancers including glioblastoma, HCC, and prostate cancer (Lee et al., 2009; Kanaoka et al., 2015; Wang et al., 2017). Juglone works by irreversibly inhibiting PIN1 catalytic domain PPIase activity and a high concentration of juglone (10–20 μM) reduced PIN1 protein expression (Hennig et al., 1998; Wang et al., 2017). In the mouse model study, intraperitoneal injection of juglone inhibits tumor growth of prostate cancer (Kanaoka et al., 2015). Although juglone is effective against cancer cell proliferation *in vitro* and *in vivo*, the lack of PIN1 specificity limits its potential use in cancer treatment. Juglone can also regulate the functions of other proteins by inhibiting RNA polymerase II and preventing the dephosphorylation of Rab4 in mitotic cells (Chao et al., 2001; Fila et al., 2008). More research is therefore required to identify or design potent and specific PIN1 inhibitors for cancer therapy.

Through screening of different chemical compound libraries, several research groups have identified specific compounds that compete for the binding to the PIN1 PPIase domain and thus inhibit PIN1 activity. Both PiB and dipentamethylene thiuram monosulfide (DTM) are found to exert anti-proliferative effect on human colon cancer cells by inhibiting PIN1 activity (Uchida et al., 2003; Tatara et al., 2009). TME-001 has also been reported to inhibit PIN1 activity and cervical cancer cell proliferation (Mori et al., 2011). An indirubin derivative (5′-nitro-indirubinoxime, 5′-NIO) has been shown to decrease PIN1 protein expression, induce G1 phase cell cycle arrest, and reduce proliferation of lung cancer cells (Yoon et al., 2012). A recent study has also reported a selenium derivative as one of the novel PIN1 inhibitors to reduce cell viability in tumorsphere formation by breast cancer stem cells (Subedi et al., 2016). Although these PIN1 inhibitors are effective anti-proliferative agents against various human cancer cells *ex vivo*, their effectiveness and toxicity in the animal models have not been formally evaluated. Moreover, poor solubility of PiB in DMSO and non-specific inhibition toward PLK1 by 5′-NIO limit their potential to be therapeutic drugs for clinical uses.

Another newly identified PIN1 inhibitor, all-*trans* retinoic acid (ATRA), is an FDA approved drug used for acute promyelocytic leukemia (APL) therapy. The PIN1-inhibitory activities of ATRA have been extensively studied *in vitro* and *in vivo* (Wei et al., 2015). Similar to other PIN1 inhibitors, ATRA inhibits PIN1 activity by directly binding to the PIN1 catalytic PPIase domain. This binding not only induces PIN1 protein degradation, but also inhibits the oncogenic functions of PIN1 by blocking PIN1-induced centrosome amplification and reducing cyclin D1 expression. In addition, ATRA-induced PIN1 downregulation results in PML-RAR-α oncoprotein degradation, leading to anti-proliferative effect against APL cells in human cell lines and mouse models. The slow-release formulation of ATRA that maintains ATRA plasma concentration in a steady level have been applied in *in vivo* studies as the short half-life of ATRA may reduce its anticancer efficacy against solid tumors. The slow-release formulation of ATRA has been shown to induce PIN1 degradation and reduce tumourigenicity in xenograft mouse model of breast cancer and HCC (Wei et al., 2015; Liao et al., 2017). Moreover, this slow-release formulation has also been reported to enhance the cytotoxic effect of other molecular targeting therapy. Combination of ATRA with sorafenib, which is a multi-kinase inhibitor for the treatment of advanced HCC, synergistically reduces PIN1 protein expression, induces cell death and inhibits tumor growth of HCC as compared with ATRA or sorafenib alone (Zheng et al., 2017). In addition to inducing PIN1 protein degradation, ATRA has been found to increase the cellular uptake of another PIN1 inhibitor, arsenic trioxide (ATO), in breast cancer cells through up-regulation of cell membrane arsenic transporter aquaporin 9 (Leung et al., 2007; Kozono et al., 2018). ATO is another FDA approved drug used for the treatment of APL patients who do not response or relapse after ATRA therapy. ATO directly binds to the PIN1 catalytic domain by a non-covalent mechanism and induces PIN1 protein degradation through the proteasome pathway (Kozono et al., 2018). As a result, ATO inhibits PIN1 activity and numerous PIN1-mediated oncogenic pathways, leading to PIN1-expressing cancer cells growth inhibition. Moreover, combination of ATO and ATRA exerts a synergistic effect in inhibiting breast cancer cell proliferation as compared with ATO or ATRA alone. In addition to PIN1, ATO can degrade other oncogenic proteins including cyclin D1 and nucleophosmin-anaplastic lymphoma kinase (NPM-ALK) (Lo and Kwong, 2014; Piao et al., 2017). All these findings suggest that both ATRA and ATO are attractive PIN1-targeting therapeutic drugs in the treatment of cancers.

A more recent study by Campaner et al. (2017) has identified a novel PIN1 inhibitor, KPT-6566, with higher potency and specificity from a huge drug library of 200,000 commercial compounds. KPT-6566 mediates PIN1 degradation, resulting in reduction of Rb phosphorylation and cyclin D1 expression. Unlike other PIN1 inhibitors that only exert anti-proliferative activity, KPT-6566 also possesses cytotoxic effects on cancer cells through generation of reactive oxygen species. KPT-6566 induces apoptosis and inhibits cell proliferation of cancer cells including breast, prostate, lung and pancreatic cancer. KPT-6566 has also been demonstrated to exert a higher anti-proliferative effect on cancer cells than normal epithelial cells. More importantly, KPT-6566 shows growth-inhibitory effects on PIN1-expressing cells but not PIN1-silenced cells, suggesting that KPT-6566 is more specific in inhibiting PIN1 activity than the aforementioned PiB that exerts growth-inhibitory effects in both PIN1-expressing and depleted cells. As for the *in vivo* study, KPT-6566 has been found to reduce the incidence of lung metastasis in mouse model of breast cancer. However, further pre-clinical and clinical studies are required to evaluate the safety and efficacy of KPT-6566 in cancer patients.

In addition to the development of more specific and potent PIN1 inhibitors, establishing specific and efficient strategies for the delivery of PIN1 inhibitors to tumor cells are equally important. Some compounds show more potent effect against PIN1 PPIase activity, but the lack of solubility limits their use for cancer treatment. A liposomal/cyclodextrin (LC) complex is designed to encapsulate a potent PIN1 inhibitor Compound 8 (Guo et al., 2009) to enhance the effectiveness of its delivery to the tumor site in a murine model (Russo Spena et al., 2018). After encapsulation in LC complex, the solubility of Compound 8 increases by 6 times. LC-Compound 8, but not the unencapsulated form, inhibits the proliferation of ovarian cancer cells *in vitro*. Similar to ATRA and KPT-6566, LC-Compound 8 induces PIN1 proteasomal degradation and inhibits numerous PIN1-mediated oncogenic pathways. More importantly, LC-Compound 8 inhibits the tumor growth of subcutaneous xenografts of ovarian cancer cells without affecting the body weight of nude mice. After intraperitoneal injection of LC-Compound 8 into nude mice bearing subcutaneous xenografts, a higher concentration of LC-Compound 8 is detected in tumor tissue than heart and liver tissues.

## Conclusion

Given its binding and isomerization activity, PIN1 regulates cell cycle progression by modulating the functions of many cell cycle-regulatory proteins. PIN1 promotes G1 checkpoint progression through a positive feedback loop comprising Rb, E2F, PIN1, and cyclin D1-CDK4/6 proteins. During S phase, PIN1 increases CDK2 activity to promote S phase progression in spite of its associated decrease and increase in cyclin E and p27 levels, respectively. Finally, PIN1 regulates various mitotic proteins to co-ordinate a proper chromosome condensation, chromatids segregation and cell division. Therefore, PIN1 over-expression leads to cell cycle deregulation and malignant cell transformation. The oncogenic functions of PIN1 make it an attractive target for cancer therapy. Several PIN1 inhibitors including ATRA and KPT-6566 have been shown to inhibit cancer cells proliferation *in vitro* and *in vivo*. Further studies are required to identify specific and non-toxic PIN1 inhibitors for the treatment of cancers with PIN1 over-expression.

## Author Contributions

All authors wrote and approved the review.

## Conflict of Interest Statement

The authors declare that the research was conducted in the absence of any commercial or financial relationships that could be construed as a potential conflict of interest.
